# Systematic thyroid screening in myotonic dystrophy: link between thyroid volume and insulin resistance

**DOI:** 10.1186/s13023-019-1019-3

**Published:** 2019-02-13

**Authors:** Adrien Ben Hamou, Stéphanie Espiard, Christine Do Cao, Miriam Ladsous, Camille Loyer, Alexandre Moerman, Samuel Boury, Maéva Kyheng, Claire-Marie Dhaenens, Vincent Tiffreau, Pascal Pigny, Gilles Lebuffe, Robert Caiazzo, Sébastien Aubert, Marie Christine Vantyghem

**Affiliations:** 1 CHU Lille, Endocrinology, Diabetology and Metabolism, F-59000 Lille, France; 2 CHU Lille, Clinical Genetics, F-59000 Lille, France; 3 CHU Lille, Radiology, F-59000 Lille, France; 4 CHU Lille, EA 2694 – Public Health, Epidemiology and Quality of Care, F-59000 Lille, France; 5 Univ Lille, Inserm, CHU Lille, UMR 837-1, Alzheimer & Tauopathies, F-59000 Lille, France; 6 CHU Lille Neuromuscular Reference Center, F-59000 Lille, France; 7 CHU Lille, Institute of Biochemistry and Molecular Biology – Biology Center, F-59000 Lille, France; 8 CHU Lille, Anesthesiology, F-59000 Lille, France; 9 CHU Lille, General and Endocrine Surgery, F-59000 Lille, France; 10 CHU Lille, Institute of Biochemistry and Molecular Biology – Pathology Center, F-59000 Lille, France; 11 Univ Lille, Inserm, CHU Lille, UMR 1190 Translational Research in Diabetes, F-59000 Lille, France; 12 EGID European Genomics Institute for Diabetes, CHU Lille, F-59000 Lille, France; 13 Department of Endocrinology, Diabetology and Metabolism, CHR-U Lille, 1, Rue Polonovski, 59037 Lille, France

**Keywords:** Myotonic dystrophy, Papillary thyroid carcinoma, Ultrasound scan, Thyroid nodule, Thyroid goiter

## Abstract

**Background:**

Myotonic dystrophy (DM1), a neuromuscular disease related to *DMPK* gene mutations, is associated to endocrine disorders and cancer. A routine endocrine work-up, including thyroid ultrasound (US), was conducted in 115 genetically-proven DM1 patients in a neuromuscular reference center. The aim of this study was to determine the prevalence and the causes of US thyroid abnormalities in DM1.

**Results:**

In the whole population (age 45.1 ± 12.2 years, 61.7% female), palpable nodules or goiters were present in 29.2%. The percentage of US goiter (thyroid volume > 18 mL) and US nodules were, respectively, 38.3 and 60.9%. Sixteen of the 115 patients had a thyroidectomy, after 22 fine-needle aspiration cytology guided by thyroid imaging reporting and data system (TIRADS) classification. Six micro- (1/6 pT3) and 3 macro-papillary thyroid carcinoma (PTCs) (2/3 intermediate risk) were diagnosed (7.9% of 115). Thyroid US led to the diagnosis of 4 multifocal and 2 unifocal (including 1 macro-PTC) non-palpable PTCs. Ultrasound thyroid volume was positively correlated to body mass index (BMI) (*p* = 0.015) and parity (*p* = 0.036), and was inversely correlated to TSH (*p* < 0.001) and vitamin D levels (*p* = 0.023). The BMI, the frequencies of glucose intolerance and PTC were significantly higher in UsGoiter versus non-UsGoiter groups.

**Conclusion:**

In this systematically screened DM1 cohort, the frequency of UsGoiter, mainly associated to BMI, was about 40%, US nodules 60%, thyroidectomies 13–14%, and PTCs 8%, two-thirds of them being micro-PTCs with good prognosis. Therefore, a systematic screening remains debatable. A targeted US screening in case of clinical abnormality or high BMI seems more appropriate.

**Electronic supplementary material:**

The online version of this article (10.1186/s13023-019-1019-3) contains supplementary material, which is available to authorized users.

## Introduction

Myotonic dystrophy (DM) is the most common inherited, autosomal dominant neuromuscular disorder in adults, affecting 1 out of 8000 people. This multi-systemic disease causes myotonia and muscle weakness in skeletal muscles with a risk of life-threatening cardiorespiratory disorders. The disease is very heterogeneous with regard to the age of onset, clinical manifestations, and severity. Two genetic types of DM have been described corresponding to an expansion of, respectively, cytosine thymidine guanine (CTG) and CCTG repeats in non-coding regions (3′-untranslated region) of the myotonic dystrophy protein kinase (*DMPK*) gene for DM1, and zinc finger protein 9 (*ZnF9*) gene for DM2.

The length of the (CTG)_n_ repeat expansion in DM1 is correlated with the severity of the disease and age of onset, defining five clinical types (congenital, infantile, juvenile, adult onset and late onset forms) [1]. The nuclear retention of mutant ribonucleic acid (RNA) alters RNA metabolism in patient’s tissues by targeting RNA-binding proteins, particularly Cytosine-Uridine-Guanine-binding protein 1 (CUGBP1) and muscle blind-like protein 1 (MBNL1). The phenotype variability is also attributed to an anticipation mechanism and possible somatic mosaicism. There is currently no curative treatment [1, 2].

Establishment of patient registries, such as DM-Scope [1–3], helps to define the phenotype of patients. DM1 patients have an increased incidence of endocrine dysfunction, especially gonadal insufficiency, diabetes and thyroid disorders [4, 5]. Palpable thyroid gland abnormalities have been described in about 20% of DM1 patients. Moreover, an increased risk of cancer [6–12], including thyroid cancer, was also reported in three recent observational studies (national Swedish/Danish, United Kingdom and American myotonic dystrophy patient registries) and one meta-analysis [13–15]. However, no studies have specifically targeted a systematic biological and imaging thyroid check-up.

The purpose of this study was to determine the prevalence of thyroid disorders, especially goiters, and any correlated factors in a French cohort of DM1 patients who received the same standardized thyroid evaluation, including a systematic thyroid ultrasound (US). We secondly aimed at identifying goitrogenic factors in this population.

## Patients and methods

### Study design and patients

**Fig. 1:**
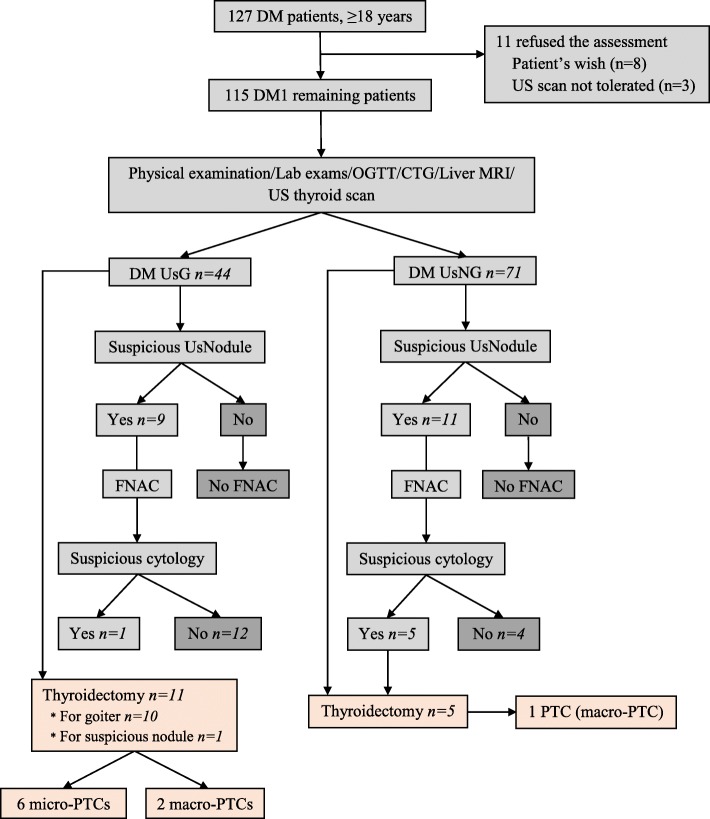
Flow chart of the study. *DM* Myotonic dystrophy, *OGTT* Oral glucose tolerance test, *MRI* Magnetic resonance imaging, *US* Ultrasound thyroid scan, *UsG* Ultrasound goiter, *UsNG* Ultrasound non-goiter, *PTC* Papillary thyroid carcinoma. We excluded the only type 2 DM patient from the analysis

This retrospective observational cohort single-center study was conducted in a university hospital setting, after approval of the hospital ethical committee (Additional file 1). Patients with DM1 that had been proven genetically, after providing a written informed consent, were systematically referred by the Neuromuscular Reference Center for a baseline multidisciplinary (neuromuscular, cardiac, pulmonary, ophthalmologic) evaluation. A routine endocrine/metabolic evaluation, including clinical, biological and US thyroid assessment was performed in the Endocrine unit. The cohort was divided into two groups, UsGoiter (ultrasound goiter) or UsNon-Goiter (ultrasound non-goiter), based on the presence or absence of a goiter, with a thyroid volume cut-off of 18 mL regardless the gender (Fig. 1).

### Patients

One hundred twenty-seven DM patients aged over 18 years were referred between 2000 and 2016 for an endocrine evaluation from the Reference Center of an area of 4 million inhabitants. Eleven patients were excluded, either due to their refusal of the evaluation (*n* = 8) or due to intolerance of the position required for the thyroid US (*n* = 3) (Fig. 1). Another one proved to be a DM2 form and was excluded. The data from the remaining 115 patients were retrospectively collected from their medical files.

### Outcomes

The following information was recorded:

- ***Clinical:*** sex, age, parity, smoking habits, family history of thyroid diseases, body mass index (BMI), cardiac and lung disorders, clinical forms according to DM-Scope [1], clinical cervical neck examination and treatment.
- ***Biological:*** thyroid function tests (TSH, FT4, FT3, thyroid peroxidase (TPO) antibodies), creatine phosphokinase (CPK), glycated hemoglobin (HbA1c), cholesterol and triglyceride levels, T0 and T120 minute blood glucose and insulin levels during an oral glucose tolerance test (OGTT) in non-diabetic patients, vitamin D measurement, and number of CTG repeats of the *DMPK* gene.
- ***Imaging:*** thyroid US.
- ***Surgical:*** number of thyroidectomies, number of micro- and macro-papillary thyroid carcinomas (PTCs).

### Biological and genetic evaluation

Laboratory tests were performed in the hospital lab with routine assay kits: TSH, anti-TPO and anti-thyroglobulin antibodies were measured with, respectively, UniCell® DxI 800 Immunoassay System (Beckman Coulter, Inc) using Access TSH 3rd IS (normal range [0.4–3.6 μIU/mL]), Access TPO antibodies (normal < 5 U/mL) and Access thyroglobulin antibodies II (normal < 0.15 ng/mL after total thyroidectomy). Diabetes and glucose intolerance were diagnosed according to the American Diabetes Association (ADA) and European Association for the Study of Diabetes (EASD) guidelines, or if antidiabetic drugs were used.

Number of CTG repeats of the *DMPK* gene was evaluated on genomic deoxyribonucleic acid (DNA) extracted from peripheral blood leukocytes, with 3 complementary assays.

### Imaging

The thyroid US evaluation was performed with linear high frequency probes (TOSHIBA Aplio XG™ SSA790A, − 9 to 13 MHz). Nodules were classified according to the Thyroid Imaging Reporting and Data System Classification (TIRADS) [16]. Fine-needle aspiration cytology (FNAC) with a 27-gauge needle was performed if nodule was: 1) TIRADS 5 and > 5 mm, 2) TIRADS 4B and > 7 mm, 3) TIRADS 4A and > 10 mm, 4) TIRADS 3 and > 20 mm. We analyzed the FNAC using the Bethesda (2010) classification.

### Statistical analyses

Normality of distribution was assessed using histograms and the Shapiro-Wilk test. Quantitative variables were expressed as mean (standard deviation) in the case of a normal distribution; otherwise the median (interquartile range) was used. Categorical variables were expressed as numbers (percentage). The percentage of patients with an US diagnosis of goiter and cancer was calculated with their 95% exact confidence intervals (CI).

Bivariate comparisons between the two study groups were made using the Student’s t-test for Gaussian continuous variables, the Mann-Whitney U test for non-Gaussian continuous variables and the Chi-squared test (or Fisher’s exact test for expected cell frequency < 5) for categorical variables, as appropriate.

In the whole DM1 group, the association of thyroid volume with patient’s characteristics was performed by using the Spearman’s rank correlation test for continuous characteristics and the Mann-Whitney U or Kruskall Wallis tests for categorical variables.

Statistical testing was done at the two-failed α level of 0.05. Data were analyzed using SAS software, version 9.4 (SAS Institute, Cary, NC, USA).

## Results

General, metabolic and thyroid characteristics of the whole cohort are given in Tables 1, 2 and 3, respectively.

**Table 1:** General characteristics of the whole group and the two subgroups according to the presence of an ultrasound goiter

Patient characteristics	Whole group	UsGoiter	UsNonGoiter	*p*-value
Number of patients *(N; %)*	*N* = 115	*N* = 44 (38.3)	*N* = 71 (61.7)	
Age *(mean ± SD, years)*	45.1 ± 12.2	46.6 ± 9.7	44.1 ± 13.5	.26
Sex				
Female ♀ *(n/N; %)*	71/115 (61.7)	28/44 (63.6)	43/71 (60.6)	.86
Minimum of one child *(n/N; %)*	45/71 (63.4)	18/28 (66.7)	27/43 (62.8)	.74
Smoking habits *(n/N; %)*	45/105 (42.9)	17/44 (38.6)	28/60 (46.7)	.36
Clinical forms *(n/N; %)*				
Congenital form	5/115 (4.3)	2/44 (4.5)	3/71 (4.2)	.88
Infantile form	14/115 (12.2)	5/44 (11.4)	9/71 (12.7)	.76
Juvenile form	21/115 (18.3)	13/44 (29.5)	8/71 (11.3)	.08
Adult form	57/115 (49.6)	31/44 (70.5)	26/71 (36.6)	.09
Late onset form	18/115 (15.6)	6/44 (13.6)	12/71 (16.9)	.10
Weight *(mean ± SD, kg)*	72.5 ± 18.0	78.2 ± 18.6	69.6 ± 16.8	**.011**
BMI *(mean ± SD, kg/m*^*2*^*)*	26.4 ± 6.5	28.1 ± 7.1	25.0 ± 5.9	**.013**
Number of CTG repeats *(median (IQR))*	500 (260–850)	500 (300–850)	515 (254–785)	.90
CPK *(median (IQR); IU/L)*	204 (108–293)	217 (106–312)	186 (112–278)	.56
Obstructive sleep apnea syndrome *(n/N; %)*	65/115 (56.5)	27/44 (61.4)	38/71 (53.5)	.55
CPAP *(n/N; %)*	40/65 (61.5)	16/27 (35.6)	24/38 (33.8)	.89
Pacemaker or defibrillator (CIED) *(n/N; %)*	28/115 (24.3)	13/44 (29.5)	15/71 (21.1)	.34

Abbreviations: *Us* Ultrasound, *SD* standard deviation, *IQR* Interquartile, *BMI* Body mass index, *CPK* Creatinine phosphokinase, *CPAP* Continuous positive airway pressure, *CIED* Cardiovascular implantable electric device, *CTG* cytosine-thymidine-Guanine

*Clinical forms*: (1) *congenital form*: neonatal (mild to severe) hypotonia, respiratory distress, sucking or swallowing difficulties, or skeletal deformities detected at birth or during the first month of life; (2) *infantile form*: clinical onset from one month to 10 years; (3) *juvenile form*: onset at 11–20 years; (4) *adult form*: onset at 21–40 years; (5) *late-onset form*: onset after 40 years

**Table 2:** Biological metabolic parameters of the whole group and the two subgroups according to the presence of an ultrasound goiter

Metabolic parameters	Whole group	UsGoiter	UsNonGoiter	*p*-value
Number of patients *(N; %)*	*N* = 115	44 (38.3)	71 (61.7)	
Glucose metabolism				
Abnormal glucose tolerance *(n/N; %)*	16/112 (14.3)	7/44 (15.9)	9/68 (13.2)	.69
Diabetes *(n/N; %)*	30/115 (26.1)	10/44 (22.7)	20/70 (28.6)	.45
Insulin therapy	3/46 (6.5)	0/17	3/29 (10.4)	**.02**
Metformin therapy	9/46 (19.6)	4/17 (23.5)	5/29 (17.2)	.12
Insulin + Metformin	3/46 (6.5)	1/17 (5.9)	2/29 (6.9)	.43
HbA1c *(median (IQR); %)*	5.5 (5.2–5.8)	5.4 (5.2–5.8)	5.5 (5.3–5.7)	.39
OGTT *(mean ± SD)*				
Glucose T0 *(mg/dL)*	90.0 ± 15.0	90.0 ± 20.0	90.0 ± 10.0	.60
Glucose T120 min *(mg/dL)*	115.0 ± 50.0	140.0 ± 50.0	90.0 ± 50.0	**.048**
Insulin T0 *(mU/L)*	7.4 ± 6.1	8.1 ± 6.9	6.6 ± 5.2	.11
Insulin T120 min *(mU/L)*	42.5 ± 37.4	45.8 ± 31.5	39.2 ± 43.2	**.051**
Lipids *(mg/dL)*				
Total cholesterol *(mean ± SD)*	200.0 ± 40.0	200.0 ± 40.0	200.0 ± 40.0	.59
LDL_c_ *(median (IQR))*	117 (96–140)	110 (90–140)	120 (100–140)	.96
HDL_c_ *(median (IQR))*	50 (43–59)	50 (40–60)	50 (40–60)	.99
Triglycerides *(median (IQR))*	132 (97–192)	140 (110–180)	130 (90–220)	.89
Vitamin D				
Median (IQR) *(ng/ml)*	17 (12–28)	17 (11–28)	18 (13–30)	.15
*n* (< 30 ng/mL)*/N (%)*	88/115 (76.5)	35/44 (79.5)	53/71 (74.7)	.51

Abbreviations: *SD* standard deviation, *IQR* interquartile, *Us* Ultrasound, *OGTT* Oral glucose tolerance test, *LDL* Low density lipoprotein, *HDL* High density lipoprotein

**Table 3:** Thyroid parameters of the whole group and the two subgroups according to the presence of an ultrasound goiter

Thyroid parameters	Whole group	UsGoiter	UsNonGoiter	*p*-value
Number of patients *(N; %)*	*N* = 115	44 (38.3)	71 (61.7)	
Family history of thyroid disorders *(n/N; %)*	14/115 (12.2)	7/44 (15.9)	7/71 (9.8)	.76
Cancer	1/115 (0.9)	1/7 (14.3)	0	NA
Clinical evaluation				
Goiter *(n/N; %)*	19/115 (16.5)	19/44 (43.2)	0	**<.001**
Nodule *(n/N; %)*	22/115 (19.1)	12/44 (27.3)	10/71 (14.1)	**<.001**
Ultrasound evaluation				
Thyroid volume *(mean ± SD, mL)*	22.9 ± 15.0	33.4 ± 25.2	12.2 ± 4.5	**<.001**
Female ♀ *(mean ± SD, mL)*	19.5 ± 19.3	30.8 ± 26.5	11.9 ± 3.5	**<.001**
Male ♂ *(mean ± SD, mL)*	22.2 ± 19.1	37.6 ± 23.1	12.7 ± 5.8	**<.001**
< 45 years old (*mean* +/- *SD*)	18.3 ± 12.3	28.6 ± 14.5	12.4 ± 4.8	**.015**
> 45 years old (*mean* +/- *SD*)	23.3 ± 25.0	38.1 ± 32.3	11.9 ± 4.2	**.003**
UsNodule > 5 mm (TIRADS) *(n/N; %)*	70/115 (60.9)	36/44 (81.8)	34/71 (47.9)	**.0006**
2	18/70 (25.7)	11/36 (30.6)	7/34 (20.6)	
3	23/70 (32.8)	9/36 (25)	14/34 (41.2)	
4 (4A and 4B)	16/70 (22.9)	7/36 (19.4)	9/34 (26.5)	
5	4/70 (5.7)	2/36 (5.6)	2/34 (5.9)	
Missing data	9/70 (12.9)	7/36 (19.4)	2/34 (5.9)	
FNAC – Bethesda (22/116 FNAC)	22/70 (31.4)	13/36 (36.1)	9/34 (26.5)	**.03**
1} Non-diagnostic	5/22 (22.8)	4/13 (30.8)	1/9 (11.1)	
2} Benign: 0–3% of malignancy	11/22 (50.0)	8/13 (61.5)	3/9 (23.1)	
3 and 4} 5–30% of malignancy	3/22 (13.6)	0	3/9 (23.1)	
5 and 6} 60–99% of malignancy	3/22 (13.6)	1/13 (7.7)	2/9 (15.4)	
Biological evaluation				
TSH *(mean ± SD; μIU/mL)*	1.7 ± 1.2	1.3 ± 0.9	2.0 ± 1.2	**<.001**
< 0.4 *(n/N; %)*	9/115 (7.8)	5/44 (11.4)	4/71 (5.6)	
0.4–3.6 *(n/N; %)*	92/115 (80.0)	34/44 (77.3)	58/71 (81.7)	
> 3.6 *(n/N; %)*	15/115 (13.0)	6/44 (13.6)	9/71 (12.7)	.74
FT4 *(mean ± SD; ng/dL)*	0.9 ± 0.3	0.9 ± 0.3	0.8 ± 0.2	**.01**
FT3 *(mean ± SD; pg/mL)*	3.4 ± 0.6	3.4 ± 0.6	3.4 ± 0.5	.77
Anti-TPO Antibodies *(n/N; %; IU/L)*				.51
Negative	52/115 (45.2)	23/44 (52.3)	29/71 (40.8)	
Positive	52/115 (45.2)	19/44 (43.2)	33/71 (46.5)	
Missing data	12/115 (10.4)	3/44 (6.8)	9/71 (21.9)	
Thyroid cancer *(n/N; %)*	9/115 (7.8)	8/44 (18.2)	1/71 (1.4)	**.002**
Recurrence (clinical and US scan)	0	0	0	
Rise of thyroglobulin	1/9 (11.1)	1/8 (12.5)	0	
Death	0	0	0	
Treatment *(n/N; %)*				NA
Total thyroidectomy – indications *(n/N; %)*	16/115 (13.9)	11/44 (25)	5/71 (7.1)	
For goiter (compression, aesthetic issues)	10/16 (62.5)	10/11 (91)	0/5	
For suspicious nodule	6/16 (37.5)	1/11 (9)	5/5 (100)	
Radioiodine therapy for carcinoma	3/9 (33.3)	2/44 (4.5)	1/71 (1.4)	
LT4 Therapy	23/115 (20)	12/44 (27.3)	11/71 (15.5)	

Abbreviations: *SD* standard deviation, *IQR* interquartile, *Us* Ultrasound, *FNAC* Fine needle aspiration cytology, *TPO* Thyroperoxydase, LT4 Levothyroxine

*Nodule classification* – Always considering the nodule with the higher TIRADS if there were multiple nodules, *FNAC* Fine needle aspiration cytology, *TPO* Thyroperoxydase auto-antibodies

### General characteristics

The mean age of the 115 patients was around 45 years, 61.7% being female, with an adult form of the disease in 49.6% of cases. Slightly more than one-third was smokers. Forty-nine percent of the patients had a body mass index (BMI) ≥25 kg/m^2^. The median number of CTG repeats was 500 (260–850). More than 50% of the cohort had an obstructive sleep apnea syndrome and one-quarter a cardiac implantable electric device (Table 1).

### Metabolic characteristics

Thirteen percent of the patients had abnormal glucose tolerance results, and one-quarter had diabetes. About one third of the patients with abnormal glucose metabolism received antidiabetic therapy. About 25% of the 115 patients received statins. A low vitamin D level (≤30 ng/mL) was observed in about three-quarters of the patients (Table 2).

### Thyroid evaluation

#### History and clinic

Twelve percent of DM1 patients had a family history of thyroid disorders. Nineteen of the 115 patients (16.5%) had a palpable goiter, and 19.1% had a palpable nodule (18 out of these 22 patients had both) (Table 3).

#### Ultrasound and cytological evaluation

Ultrasound examination showed that 38.3% of the 115 patients, including 61.7% female, had a thyroid volume > 18 mL in favor of goiter, defining the UsGoiter group. The 71 remaining patients with normal thyroid volume constituted the UsNon-Goiter group. There was no difference of thyroid volume according to age (cut-off 45 years) or sex in the whole group.

Sixty-one percent (*n* = 70/115) of the patients had at least one ultrasound nodule (> 5 mm) (Table 3 and Fig. 1), and 20 of the 70 (28.6%) had at least one suspicious nodule, classified as TIRADS 4A and B (*n* = 16/115 patients [13.9%]), 50% of which were palpable, or TIRADS 5 (*n* = 4/115 [3.5%]), 75% of which were palpable.

Twenty-two FNAC were conducted in these 20 patients with the following results: 22.7% had a non-diagnostic cytology (Bethesda I), 50% had a benign cytology (Bethesda II) and 27.2% had indeterminate cytology including three Bethesda 3 or 4 (meaning 5 to 30% of malignancy risk), and three cases of Bethesda 5 or 6 (meaning 60 to 99% of malignancy risk).

#### Biological evaluation

The mean TSH, FT4 and FT3 levels of the whole group were in the reference range. Nevertheless, 7.8% of the patients had a TSH level below the lower limit and 13% above the upper limit of reference range. These patients with thyroid dysfunction did not have medical or surgical treatment for thyroid disease. Fifty percent of the patients had positive (above the upper limit of normal range) anti-TPO antibody levels. However, there was no association between thyroid volume and antibodies blood levels (Table 3).

#### Treatment for thyroid dysfunction or thyroid dystrophy

One patient received a dose of radioiodine for a toxic thyroid nodule. During the follow-up, 23/115 patients (20%) needed LT4 therapy for hypothyroidism, not taking into accounts those who had a thyroidectomy.

Sixteen patients out of the cohort (11 UsGoiter and 5 UsNon**-**Goiter patients) underwent total thyroidectomy (15 by cervicotomy and 1 by robotic trans-axillary thyroidectomy), either because the results of the FNAC were suspicious for malignancy per the Bethesda classification (*n* = 1 in the UsGoiter group and *n* = 5 in the UsNon**-**Goiter group) or because of the size (causing compression in 7 cases) of the goiter (*n* = 10) (Fig. 1). Indeed eight out of the 115 patients (6.9%) had a thyroid volume ≥ 40 mL.

Three patients had lymph node dissections, including both a central and lateral neck lymph node dissection (*n* = 1), a central lymph node dissection (*n* = 2) and a “node-picking” lymphadenectomy (*n* = 1). One patient had a difficult endotracheal intubation that had been predicted by the intubation difficulty scale (IDS). All operated DM1 patients spent their first post-operative night in the intensive care unit as a precaution. No serious post-anesthetic or post-operative complications were reported (e.g., no recurrent paralysis or postoperative hypocalcemia).

#### Thyroid carcinomas

Nine cases of PTC (55.6% men) were diagnosed out of the 16 thyroidectomies, at a mean age of 48.7 ± 7.0 years. Six were papillary micro-carcinomas (micro-PTCs), aged between 41 and 53 years, all found in the UsGoiter group, and 3 were well-differentiated macro-PTCs (≥1 cm), aged between 48 and 60 years with only one, having low risk, in the UsNon-goiter group. Four were multifocal carcinomas, including 3 macro-carcinomas. Two macro-carcinomas (follicular variant of PTC) had an intermediate risk of recurrence in the UsGoiter group (pT2_(m)_N1_a_ with more than 5 lymph nodes invaded and pT3_(m)_N0). One micro-carcinoma had an extra-capsular extension (pT3_(s)_N0). Only 2 patients with macro-carcinomas had lymph node metastasis (central location – N1_a_). None of the patients had other metastasis. Three adjuvant radioiodine ablations (RIA) were performed and one patient refused RIA because of the myotonic stage and asthenia. Of the 9 patients with thyroid cancer, four patients (age 42.7 ± 7.8 years) had six other neoplastic lesions, including 1 sclerodermiform basocellular skin carcinoma (diagnosed at 38 years), 1 basocellular skin carcinoma (diagnosed at 54 years), 1 giant prolactinoma (diagnosed at 49 years), 1 pleomorphic adenoma of the parotid gland (diagnosed at 41 years), one insulinoma associated to a non-secreting neuroendocrine pancreatic tumor, and 1 pilomatrixoma (diagnosed at 30 years). All the patients had a long-term follow-up (median of 5 years) with negative thyroglobulin levels; the exception was one patient (pT2_(m)_N1_a_) who showed a very mild increase in thyroglobulin (1.91 ng/mL; normal after treatment < 0.15 ng/mL) without thyroglobulin antibodies, despite previous radioiodine therapy. No ultrasound scan abnormalities were visible. A repeat thyroglobulin test and US evaluation are scheduled.

### Associations and correlations between thyroid volume and continuous variables in the whole group

Thyroid volume was positively correlated with BMI (*r* = 0.24, *p* = 0.015) and parity (*r* = 0.21, *p* = 0.036), and was inversely correlated with vitamin D levels (*r* = − 0.18, *p* = 0.023), TSH level (*r* = − 0.42, *p* < 0.0001) and FT4 level (*r* = − 0.32, *p* = 0.04).

There was no correlation between thyroid volume and age, the number of CTG repeats, smoking habits, thyroid peroxidase antibodies, HbA1c, OGTT parameters, and lipid parameters. The median number of CTG repeats tended to be higher in the cancer group than in the whole group (800 (350–1300) vs. 500 (260–800), but the difference was not significant (*p* = 0.35).

In the whole cohort, BMI was positively correlated with the number of CTG repeats (*r* = 0.352, *p* = 0.028) and was inversely correlated with vitamin D levels (*r* = − 0.269, *p* = 0.009). The number of CTG repeats was not correlated with vitamin D levels.

### Comparison of UsGoiter and UsNon-goiter groups

#### Clinical characteristics

Body weight and BMI were significantly higher in the UsGoiter than in the UsNon**-**Goiter group (*p* = 0.011 and *p* = 0.013, respectively) (Table 1). There were no differences between both groups regarding other characteristics of patient, especially the clinical DM1 forms. Moreover, the percentage of patients > 45 years and the sex ratio did not differ in each of the 2 groups (*p* = 0.71 and *p* = 0.15, respectively).

#### Biological metabolic parameters

Glucose (*p* = 0.048) and insulin (*p* = 0.051) levels at 120 min post-OGTT were or tended to be significantly higher in the UsGoiter group than in the UsNon-Goiter group (Table 2). The other metabolic parameters did not differ between the two groups.

#### Thyroid evaluation

As expected, the frequency of a palpable goiter (*p* < 0.0001), a palpable nodule (*p* < 0.0001), an US nodule (*p* = 0.0006) and the number of FNAC (*p* = 0.03) were significantly higher in the UsGoiter than in the UsNon**-**Goiter group. Significantly lower TSH (*p* < 0.001) and higher FT4 (*p* = 0.01) (though still in the reference range) levels were observed in the UsGoiter group compared with the UsNon**-**Goiter group. There was no difference between the two groups with regard to FT3 levels, the frequency of positive anti-TPO antibodies and the initially high TSH levels. More thyroid carcinomas were diagnosed in the UsGoiter group (8 carcinomas – 18.2%) than in the UsNon**-**Goiter group (1 carcinoma – 1.4%) (*p* = 0.002) (Table 3).

There was no difference between frequency of palpable goiter or nodule, US thyroid volume, PTC frequency, and number of CTG repeats between males and females (Additional file 1: Table S1).

## Discussion

This study is the first evaluation of the frequency of thyroid disorders in a cohort of DM1 patients using well-standardized ultrasound and biological evaluation. Sixty percent of this cohort of 115 patients had US thyroid nodules or goiter, 7.8% had PTCs and about 20% had sub-clinical thyroid dysfunction before any surgery.

This prevalence of 60% of thyroid dystrophy is close to the prevalence found by US in the general population (45 to 67%) [17], age-matched general population (42%), and in autopsy series (60%) [18, 19]. Thyroid volume did not differ between gender and age (more or less than 45 years) in this cohort.

The main interest of US scans was to identify nodules suspicious for malignancy according to the TIRADS classification, in a population in which a higher risk of cancer has been identified [12]. In this series, 17.4% (20/115) of the patients had suspicious nodules, classified as TIRADS 4 or 5, by US examination. About 22% of the performed FNAC led to undetermined cytology (Bethesda I) that seems relatively high compared to literature [20]. This might be related to the fact that FNAC was performed in nodules below 10 mm when TIRADS results were suspicious since the study was undertaken before American Thyroid Association (ATA) guidelines [21]. A little less than 14% of the whole cohort had a thyroidectomy because of suspicious FNAC or large goiter. A high prevalence of PTCs (nearly 8% of the cohort) was identified, which is consistent with the high frequency of cancers (especially thyroid cancer) already noted in four observational DM cohorts [11, 13–15]. The overall PTC prevalence in our cohort screened routinely by US was, however, a little higher than that previously found in other DM cohort (8% vs. about 4%), which is not surprising when considering the systematic screening effect. Indeed, the increase of PTC was mainly due to an increase of micro-PTCs, which corresponds to two-thirds of the PTCs cases in our series. A high frequency of micro-PTCs has already been reported in the general population and is attributed to an improvement in diagnostic techniques [22]. Nevertheless, to get a local reference, the prevalence of micro-PTCs in operated non-DM patients in the same surgery department, studied by the same pathologists during the same period was 17% which is lower than the prevalence observed in our operated DM1 patients (6 micro-PTCs out of 16 surgeries in the DM group, e.g., 37%). Even if this comparison should be taken with caution, it suggests that DM patients may have an increased risk of micro-PTCs, in accordance with recent data demonstrating a high frequency of thyroid cancer, in DM, for yet unknown reasons.

The relevance of routine thyroid ultrasound in DM has however to be discussed. Indeed, most cancers were micro-PTC with a good prognosis and low risk of recurrence [21]. In our series, five cancers had not been suspected clinically but were only found on US examination. The final diagnosis was two cases of micro-PTCs and one low-risk macro-PTC, and a delayed diagnosis probably would not have changed the patient’s prognosis. Nevertheless, out of the nine PTCs, four micro-PTCs were multifocal, with one of the four having a capsular breach (pT3) and two macro-PTCs were of intermediate risk. Thyroid US screening is non-invasive and not very expensive. Therefore, on one hand, routine thyroid evaluation seems justified in order to not miss any cancers. On the other hand, an over-diagnosis of PTC, which usually has a good prognosis and progresses slowly, could lead to over-treatment with all its implied risks [23] (e.g., general anesthesia and cervicotomy), especially if not done in a reference center. Most of these patients have comorbidities and are considered high-risk in terms of cardiorespiratory complications, although no serious postoperative complications were reported in our group. Follow-up study is needed to determine if the evolution pattern of PTCs in DM patients and also the prognosis are similar to general population. This will help to justify a systematic screening, in this population, now considered as a high-risk population for cancer.

Our secondary aims were to determine whether any associated factors to goiter in this DM population, could guide the US thyroid screening. The classical factors of goitrogenesis, such as family history of thyroid disease and smoking, were not overtly involved, despite it must be noticed that one third of the population was smokers [24]. Female gender was more frequent in the whole cohort but was similar between the UsGoiter and UsNon-Goiter groups (63.6% vs. 60.6%). Nevertheless, childbearing was more frequent in the UsGoiter group, and the thyroid volume was associated with parity, as usually reported [25]. Patients with PTC were, however, more often male but the difference of PTCs frequency between male and female was not statistically significant in accordance with a recent meta-analysis [12]. However, a more severe phenotype in males has already been recognized in DM1 patients [3], and if the difference of number of CTG repeats between sex was not significant, it tended to be slightly higher in men as compared to females.

The most significant parameters associated with thyroid volume were BMI and glucose metabolism. This relationship has been emphasized in the literature, both in obese people [17, 26] and in normal-weight, hyperinsulinemic, lipodystrophic patients [27]. Indeed, half of our DM patients were overweight, 39% were glucose intolerant or diabetic, and 25% were receiving statins; this confirms the frequency of insulin resistance confirmed by higher 120-min OGTT glucose and insulin levels [28]. This high prevalence of metabolic abnormalities has been demonstrated in other DM cohorts [29], with a range between 14.6 and 21.1% in 1856 patients, with no difference between genders. Insulin is a growth factor and could therefore favor thyroid growth, as demonstrated in a recent age-matched control study in DM1 [30].

TSH levels were lower in the UsGoiter group compared with the UsNon**-**Goiter group, in contradiction with what would have been expected due to the goitrogenic effect of TSH. Unexpectedly, thyroid volume was inversely correlated with TSH and FT4 levels. This unforeseen result could be explained by the relative autonomy of some goiters or pituitary dysfunction related to DM1, but also suggests other promoting factors. Iodine deficiency has nearly vanished since the systematic supplementation of salt [31] in France and so was not specifically studied in our routinely screened cohort. Previous studies [32, 33] reported an association between vitamin D levels and (CTG)_n_ expansion size or anti-TPO antibodies that we did not observed in our cohort. However, we observed an inverse correlation between thyroid volume and vitamin D level. Such a correlation has never been previously described. Nevertheless, vitamin D is involved in cell differentiation and apoptosis, and a lack of vitamin D could favor goiter and/or PTC [34]. Compared to age-matched controls, DM patients have lower vitamin D levels [30], a fact we confirm since 76.5% of our population had vitamin D deficiency. This low level of vitamin D could be explained by the physical disability potentially resulting in less outdoor exposure and a higher BMI. Indeed, in our study, BMI was positively correlated to the number of CTG repeats and was inversely correlated to vitamin D deficiency, which is in accordance with some studies [35]. This supports an indirect mechanism linking vitamin D levels and thyroid volume. Even if vitamin D supplementation is recommended, no interventional studies have demonstrated that it could reduce the prevalence of goiter in this population, whatever the direct or indirect mechanisms.

Surprisingly, no correlation between thyroid abnormalities and CTG repeats was observed in our patients, despite the association between the number of CTG repeats and disease severity [1]. This is probably related to the fact that the half of our cohort corresponded to an adult form with a number of CTG repeats between 280 and 1000. Repeats size is only modestly associated with disease severity in adult-onset DM1, whose repeats size span a wide spectrum, at difference with forms associated to less than 100 or more than 1000 CTG repeats. However the median number of CTG_(n)_ repeat tended to be higher in patients who developed PTCs compared to others patients which is another argument for a higher risk related to the disease and not only a screening effect. The mechanisms leading to a higher risk of cancer in DM1 patients have not been described yet. A direct role in carcinogenesis of the abnormal RNA processing or/and the metabolic syndrome observed in these patients may contribute to increased cancer risk, as well as insulin resistance. The presence of genetic mosaicism and variable expression of CTG in thyroid tissue [36] may explain that some patients with high number of CTG repeats detected in leukocytes do not developed thyroid abnormalities.

Finally, the answer to the question “Should we screen DM1 patients by US thyroid?” remains unclear. The fact that these PTC were frequent (8% of patients), occurred at any age, were multifocal in 4/9 cases, with one third being pT3 or of intermediate risk argue for a systematic US screening. However, we did not find a higher prevalence of US goiter and nodules in this DM1 cohort compared to US-screened general population. Nevertheless, the limited size of our cohort, the fact that this cohort corresponds to a limited sample of the DM1 population followed in a reference center (probably only one third), the effect of genetic screening in pre-symptomatic patients in family inquiries, the lack of matched control group introduce selection biases. Moreover the good prognosis of most thyroid cancers identified in our series does not argue for a systematic screening. Therefore, a targeted screening in case of palpable nodule, especially in male overweight or diabetic people, seems more appropriate. If surgery is required, it should be performed by an experienced anesthesiologist and surgical team, after cardiorespiratory work-up, with a reinforced post-operative monitoring.

## Conclusion

In this cohort of DM1 patients, we observed a high prevalence of PTCs that may not be only due to the effect of a systematic screening, in accordance with the high risk of cancer described recently in DM patients. In addition, we show that these PTC were mainly micro-PTC and that overweight and glucose intolerance were the main factors associated with higher thyroid volume. Considering the good prognosis of PTCs in general population, targeted US screening in case of clinical abnormality and/or in overweight (BMI ≥ 25) or “metabolic” patients seem currently more appropriate than systematic US screening. Further studies are needed to understand the mechanism leading to cancer in DM1 patients and to study the evolution of these cancers compared to the general population. The link with insulin resistance could open new perspectives even in general population.

## Additional file

Additional file 1: Main characteristics according to gender. Values expressed as n/N (%), mean ± standard deviation (SD) or median (interquartile range). NA: not applicable. (DOCX 15 kb)

## Abbreviations

ADA: American Diabetes Association
ATA: American Thyroid Association
BMI: Body Mass Index
CI: Confidence Intervals
CPK: Creatinine Phosphokinase
CUGBP1: CUG-Binding Protein 1
DM: Myotonic Dystrophy
DM1: Myotonic Dystrophy Type 1
DMPK: Myotonic Dystrophy Protein Kinase
DNA: Deoxyribonucleic Acid
EASD: European Association for the Study of Diabetes
FNAC: Fine Needle Aspiration Cytology
FT3: Free T3
FT4: Free T4
IDS: Intubation Difficulty Scale
LT4: L-Thyroxine
MBNL1: Muscle Blind-Like Protein 1
OGTT: Oral Glucose Tolerance Test
PTC: Papillary Thyroid Carcinoma
RIA: Radioiodine Ablations
RNA: Ribonucleic Acid
TIRADS: Thyroid Imaging Reporting and Data System
TPO: Thyroid Peroxidase
TSH: Thyroid Stimulating Hormone
UK: United Kingdom
US: Ultrasound
US/USA: United States (of America)
ZnF9: Zinc Finger Protein 9
