# Supplementary material for: Systematic thyroid screening in myotonic dystrophy: link between thyroid volume and insulin resistance
Source: Orphanet J Rare Dis. 2019 Feb 13;14:42. doi: 10.1186/s13023-019-1019-3 (PMC6375124; doi:10.1186/s13023-019-1019-3)
Supplement: Supplementary file 1 — Main characteristics according to gender. Values expressed as n/N (%), mean ± standard deviation (SD) or median (interquartile range). NA: not applicable. (DOCX 15 kb) [file 13023_2019_1019_MOESM1_ESM.docx]

Additional file 1: Table S1. Main characteristics according to gender

| Parameters | Whole group  N = 115 (100) | Gender | | P-value |
| --- | --- | --- | --- | --- |
| Number of patients *(N; %)* |  | Male ♂  N = 45 (39.1) | Female ♀  N = 70 (60.9) |  |
| Number of CTG(n) *(median (IQR))* | 500 (260 – 850) | 570.0 (330.0 ; 850.0) | 475.0 (200.0 ; 700.0) | .15 |
| Clinical evaluation |  |  |  |  |
| Goitre *(n/N; %)* | 19/115 (16.5) | 8/19 (42.1) | 11/19 (57.9) | .75 |
| Nodule *(n/N; %)* | 22/115 (19.1) | 10/22 (45.5) | 12/22 (54.5) | .98 |
| Thyroid volume *(mean ± SD)* | 22.9 ± 15.0 | 22.2 ± 19.1 | 19.5 ± 19.3 | .55 |
| TSH |  |  |  |  |
| < 0.4 *(n/N; %)* | 9/115 (7.8) | 3/9 (33.3) | 6/9 (66.7) | NA |
| > 3.6 *(n/N; %)* | 15/115 (13.0) | 6/15 (40) | 9/15 (60) | NA |
| Thyroid cancer *(n/N; %)* | 9 (7.8) | 5/9 (11.1) | 4/9 (5.6) | .31 |

Values expressed as n/N (%), mean ± standard deviation (SD) or median (interquartile range). NA: not applicable.
